# A toolbox for the fast information analysis of multiple-site LFP, EEG and spike train recordings

**DOI:** 10.1186/1471-2202-10-81

**Published:** 2009-07-16

**Authors:** Cesare Magri, Kevin Whittingstall, Vanessa Singh, Nikos K Logothetis, Stefano Panzeri

**Affiliations:** 1 Italian Institute of Technology, Department of Robotics, Brain and Cognitive Sciences, I-16163 Genoa, Italy; 2 Max Planck Institute for Biological Cybernetics, D-72076 Tübingen, Germany; 3 Imaging Science and Biomedical Engineering, University of Manchester, Manchester M13 9PT, UK

## Abstract

**Background:**

Information theory is an increasingly popular framework for studying how the brain encodes sensory information. Despite its widespread use for the analysis of spike trains of single neurons and of small neural populations, its application to the analysis of other types of neurophysiological signals (EEGs, LFPs, BOLD) has remained relatively limited so far. This is due to the limited-sampling bias which affects calculation of information, to the complexity of the techniques to eliminate the bias, and to the lack of publicly available fast routines for the information analysis of multi-dimensional responses.

**Results:**

Here we introduce a new C- and Matlab-based information theoretic toolbox, specifically developed for neuroscience data. This toolbox implements a novel computationally-optimized algorithm for estimating many of the main information theoretic quantities and bias correction techniques used in neuroscience applications. We illustrate and test the toolbox in several ways. First, we verify that these algorithms provide accurate and unbiased estimates of the information carried by analog brain signals (i.e. LFPs, EEGs, or BOLD) even when using limited amounts of experimental data. This test is important since existing algorithms were so far tested primarily on spike trains. Second, we apply the toolbox to the analysis of EEGs recorded from a subject watching natural movies, and we characterize the electrodes locations, frequencies and signal features carrying the most visual information. Third, we explain how the toolbox can be used to break down the information carried by different features of the neural signal into distinct components reflecting different ways in which correlations between parts of the neural signal contribute to coding. We illustrate this breakdown by analyzing LFPs recorded from primary visual cortex during presentation of naturalistic movies.

**Conclusion:**

The new toolbox presented here implements fast and data-robust computations of the most relevant quantities used in information theoretic analysis of neural data. The toolbox can be easily used within Matlab, the environment used by most neuroscience laboratories for the acquisition, preprocessing and plotting of neural data. It can therefore significantly enlarge the domain of application of information theory to neuroscience, and lead to new discoveries about the neural code.

## Background

Information theory [1,2], the mathematical theory of communication, has been successfully used to address a number of important questions about sensory coding [3-5]. For example, information theoretic tools have been used to characterize the stimulus selectivity of neurons, by revealing the precise sensory features (or combinations of them) which modulate most reliably the responses of neurons or of neural populations [6-8]. Information theory has been used to investigate whether the fine temporal structure of neural activity contains important information which is lost when neural signals are averaged over longer time scales. These studies have shown that the precise timing of spikes measured with respect to the stimulus onset [5,9-12], or the relative time of spikes with respect to the ongoing network fluctuations [13,14] provides important information that cannot be extracted from the spike count. Information theory has also been used to study population codes: several studies have developed measures to quantify the impact of cross-neuronal correlations in population activity [15-19] and have applied them to the study of population coding across various sensory modalities [20-22]. Another application of information theory to neuroscience is the measure of causal information transfer [23] to quantify the amount of interactions between neural populations [24].

Information theory has been used widely for the analysis of spike trains from single neurons or from small populations [3], and is now beginning to be applied systematically to other important domains of neuroscience data analysis, such as the information analysis of analog brain signals like BOLD fMRI responses [25-27] and Local Field Potentials (LFPs) [28-30]. However, the application of information theory to analog brain signals has remained relatively limited compared to the potentials it offers. One reason that has limited a wider use of the information analysis to analog signals is that estimates of information from real neurophysiological data are prone to a systematic error (bias) due to limited sampling [31]. The bias problem can be alleviated by the use of several advanced techniques [5,32-36] or by combinations of them [31]. However, a detailed implementation and testing of these (often complicated) techniques is in many cases beyond the time resources available to experimental laboratories. Moreover, the performance of bias correction techniques was so far tested thoroughly primarily on spike trains, and much less on analog brain signals. Since the performance of bias correction techniques depends on the statistics of the data [31], it is crucial that tests and comparisons of the performance of the various techniques are also carried out on analog brain signals. Clearly, the availability of a toolbox that implements several of these accurate information calculation techniques after having validated them on analog brain signals would greatly increase the size of the neuroscience community that uses information theoretic analysis.

Another problem, which is particularly prominent when computing information quantities for multiple parallel recordings of neural activity, is the speed of computation. Multielectrode recordings now allow the simultaneous measurement of the activity of tens to hundreds of neurons, and fMRI experiments allow a broad coverage of the cerebral cortex and recording from a large number of voxels. Therefore speed of calculation is paramount, especially in cases when information theory is used to shed light on the interactions between pairs or small groups of recorded regions. In fact, the number of these subgroups (and thus the time needed to compute their information or interaction) increases fast with the number of recorded regions.

This article aims at meeting the demand for fast and publicly available routines for the information theoretic analysis of several types of brain signals, by accompanying and documenting the first release of the *Information Breakdown ToolBox *(ibTB). This toolbox can be downloaded from the URL  or from the Additional Files provided with this Article (Additional file 1: ibtb.zip). The toolbox has several key features that make it useful to the neuroscience community and that will widen the domain of application of information theory to neuroscience. In particular *(i) *it can be used within Matlab (The Mathworks, Natick, MA), one of the most widely used environments for the collection, preprocessing and analysis of neural data, *(ii) *it is algorithmically optimized for speed of calculation *(iii) *it implements several up-to-date finite sampling correction procedures *(iv) *it has been thoroughly tested for the analysis of analog neural signals such as EEGs and LFPs *(v) *it implements the information breakdown formalisms [17,18,37] that are often used to understand how different groups of neurons participate in the encoding of sensory stimuli.

### Definitions and meaning of neural Entropies and Information

Before proceeding to describe the implementation of the Toolbox and to discuss its use, in the following we will briefly define the basic information quantities and describe their meaning from a neuroscientific perspective.

Consider an experiment in which the experimental subject is presented with *N*_*s *_stimuli *s*_1_,... and the corresponding neural response ***r ***is recorded and quantified in a given post-stimulus time-window. The neural response can be quantified in a number of ways depending on the experimental questions to be addressed and on the experimenter's hypotheses and intuition. Here, we will assume that the neural response is quantified as an array ***r ***= [*r*_1_,..., *r*_*L*_] of dimension *L*. For example, the experimenter may be recording the spiking activity of *L *different neurons and may be interested in spike count codes. In this case *r*_*i *_would be the number of spikes emitted by cell *i *on a given trial in the response window. Alternatively, if the experimenter is recording the spiking activity of one neuron and is interested in spike timing codes, the response could be quantified by dividing the post-stimulus response window into *L *bins of width Δ*t*, so that *r*_*i *_is the number of spikes fired in the *i*-th time bin [33]. Or, the experimenter may be recording LFPs from *L *channels and be interested in how their power carries information. In this case *r*_*i *_would be quantified as the LFP power in each recording channel [29].

Unless otherwise stated, we will assume that the neural response in each element of the response array is discrete. If the signal is analogue in nature – such as for LFP or EEG recordings – we assume it has been discretized into a sufficient number of levels to capture the most significant stimulus-related variations. For example, the power at *L *distinct frequencies extracted from a single-trial LFP recording will be quantized, for each frequency, into a finite number of levels [29]. This assumption is (unless otherwise stated) necessary for the analysis and the correct functioning of the algorithms in the toolbox. The reason why it is convenient to quantify the neural response as a discrete variable is that it makes it easier to quantify the probabilities necessary for information calculation (see below). For analog signals, the discretization can be circumvented only when there are suitable analytical models for the probability distribution (*e.g*. a gaussian distribution), in which case particular algorithms can be applied (see *e.g*. the Gaussian Method detailed below).

Having defined the response, we can quantify how well it allows us to discriminate among the different stimuli by using Shannon's mutual information [1,2]:

(1)

The first term in the above expression is called the *response entropy*. It quantifies the *overall *variability of the response and is defined as

(2)

*P*(***r***) being the probability of observing ***r ***in response to any stimulus. The second term, called *noise entropy*, quantifies the response variability specifically due to "noise", *i.e*. to trial-to-trial differences in the responses to the same stimulus. *H*(***R***|*S*) is defined as

(3)

where *P*(***r***|*s*) is the probability of observing response ***r ***when stimulus *s *is presented; *P*(*s*) is the probability of presentation of stimulus *s*, and is defined as , *N*_*tr*_(*s*) being the number of trials available for stimulus *s *and  is the total number of trials to all stimuli. Mutual information quantifies how much of the information capacity provided by stimulus-evoked differences in neural activity is robust to the presence of trial-by-trial response variability. Alternatively, it quantifies the reduction of uncertainty about the stimulus that can be gained from observation of a single trial of the neural response.

The mutual information has a number of important qualities that make it well suited to characterizing how a response is modulated by the stimulus. These advantages have been reviewed extensively [3-5,25,27,38]. Here we briefly summarize a few key advantages. First, as outlined above, it quantifies the stimulus discriminability achieved from a single observation of the response, rather than from averaging responses over many observations. Second, *I*(*S*; ***R***) takes into account the full stimulus-response probabilities, which include all possible effects of stimulus-induced responses and noise. Thus, its computation does not require the signal to be modeled as a set of response functions plus noise and can be performed even when such decomposition is difficult. Third, because information theory projects all types of neural signals onto a common scale that is meaningful in terms of stimulus knowledge, it is possible to analyze and combine the information given by different measures of neural activity (for example: spike trains and LFPs) which can have very different signal to noise ratios.

### The contribution of correlations between different neural responses to the transmitted information

Neural signals recorded from different sites are often found to be correlated. For example, spikes emitted by nearby neurons are often synchronous: the probability of observing near-simultaneous spikes from two nearby neurons is often significantly higher than the product of the probabilities of observing the individual spikes from each neuron [39-41]. LFPs recorded from different electrodes are typically correlated over considerable spatial scales [42,43]. Moreover, neural signals can be correlated in time as well as in space. In fact, different temporal aspects of the neural activity from the same location are often correlated. For example, spike counts and latencies covary in some systems [44], and the powers of different LFPs bands recorded from the same location can also exhibit significant correlations [29].

The ubiquitous presence of correlations of neural activity across both space and time has raised the question of what is the impact of this correlation upon the information about sensory stimuli carried by a combination of distributed sources of neural activity (see [16,45] for recent reviews). Theoretical studies have suggested that correlations can profoundly affect the information transmitted by neural populations [16,46]. It is therefore of great interest to quantify the impact of correlations on the information carried by a population of simultaneously recorded neurons. Mutual information, Eq. (1), can tell us about the total information that can be gained by simultaneously observing the *L *considered neural responses, but not about the specific contribution of correlations to this total value. However, a number of information-theoretic quantities have been developed to quantify how correlations affect the information carried by different neural signals (see below). In our information toolbox we have implemented several of such approaches, which will be briefly define and summarize in the rest of this section.

#### Different types of correlations affecting information

Before we describe the information theoretic tools for quantifying the impact of correlations on coding, it is useful to briefly define the types of correlations usually considered in the studies of neural population activity.

The most commonly studied type of correlation of neural activity is what is traditionally called "noise correlation", that is the covariation in the trial-by-trial fluctuations of responses to a fixed stimulus [16,47]. Because these noise covariations are measured at fixed stimulus, they ignore all effects attributable to shared stimulation. Although we will stick with the well-established "noise" terminology, we point out that the name is potentially misleading since noise correlations can reflect interesting neural effects. Mathematically speaking, we say that there are noise correlations if the simultaneous joint response probability *P*(***r***|*s*) at fixed stimulus is different from the "conditionally independent" response probability

(4)

The conditional probability *P*_*ind*_(***r***|*s*) can be computed by taking the product of the marginal probabilities of individual elements of the response array, as in Eq. (4), or alternatively by the empirical "shuffling" procedure described as follows. One generates a new set of shuffled responses to stimulus *s *by randomly permuting, for each element of the response array, the order of trials collected in response to the stimulus *s *considered, and then joining together the shuffled responses into a shuffled response array. This shuffling operation leaves each marginal probability *P*(*r*_*j*_|*s*) unchanged, while destroying any within-trial noise correlations. The distribution of shuffled responses to a given stimulus *s *is indicated by *P*_*sh*_(**r**|*s*).

Many authors further distinguish noise correlations (which, as explained above, exclude sources of correlations due to shared stimulation) from "signal correlations" [47], which are correlations entirely attributable to common or related stimulus preferences. Signal correlations manifest themselves in similarities across stimuli between the response profiles of the individual elements of the response array. For example, neurons in different channels may all have a very similar mean response to the stimuli. There are several ways to quantify the amount of signal correlations [17,37,47]. We will not report them in this Article, but we will only focus on quantifying their impact on the information carried by the response array (see next section).

The importance of separating noise from signal is, as revealed by theoretical studies, that signal and noise correlations have a radically different impact on the sensory information carried by neural populations (see below). In particular, signal correlations always reduce the information, whereas noise correlations can decrease it, increase it or leave it unchanged, depending on certain conditions [46,48].

#### Information Breakdown

We next describe and define briefly several mathematical techniques to quantify the impact of correlations of information. Several different approaches have been proposed (see *e.g*. [17,18,21,49,50]). Here we present one, called the "information breakdown" [17], which takes the total mutual information *I*(*S*; ***R***) and decomposes it into a number of components, each reflecting a different way into which signal and noise correlations contribute to information transmission. We decided to focus on the information breakdown formalism partly because it was developed by one of the authors of this article, and partly because it naturally includes several of the quantities proposed by other investigators [18,21].

The information breakdown writes the total mutual information into a sum of components which are related to different ways in which correlations contribute to population coding [17], as follows:

(5)

**Figure 1:**
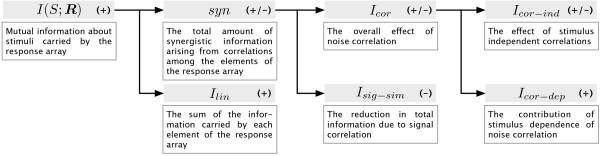
**Components of the information breakdown**. This figure shows a schematic representation of the terms of the information breakdown of Ref. [17]. The information breakdown takes the joint mutual information *I*(*S*; ***R***) and breaks it down into the sum of two terms: *I*_*lin *_(the sum of information carried by each element of the response array) and *syn *(the amount of synergistic information). The synergy can be further broken down into the terms *I*_*sig*-*sim *_and *I*_*cor *_which highlight the effect of different modes of correlation. *I*_*sig*-*sim *_quantifies the impact of signal correlations on the total information, while *I*_*cor *_quantifies the effect of noise correlation. The term *I*_*cor *_is finally broken down into *I*_*cor*-*ind *_and *I*_*cor*-*dep*_, which describe the effects of stimulus-independent and stimulus-dependent correlations respectively.

The meaning and mathematical expression of each of the components is summarized in Figure 1 and is described in the following.

*The linear term I*_*lin *_is the sum of the information provided by each element of the response array. This is a useful reference term because if all the elements of the array were totally independent (*i.e*. with both null noise and signal correlation) then the total information transmitted by the response array would be equal to *I*_*lin*_.

*The amount of synergistic information Syn*. The difference between *I*(*S*; **R**) and *I*_*lin *_is called synergy. Positive values of synergy denote the presence of synergistic interaction between elements of the response array, which make the total information greater than the sum of that provided by each element of the response array. Negative values of synergy (called "redundancy") indicate that the elements of the response array carry similar messages, and as a consequence information from the response array is less than the sum of the information provided by each individual element. The synergy can be further broken into the contributions from signal and from noise correlations, as follows.

*The signal similarity component I*_*sig*-*sim *_is negative or zero and quantifies the amount of redundancy specifically due to signals correlation. We note that the negative of *I*_*sig*-*sim *_equals the quantity named Δ*I*_*signal *_which was defined in Ref. [18].

*The noise correlation component I*_*cor *_quantifies the total impact of noise correlation in information encoding. Originally introduced in [51], it equals the difference between the information *I*(*S*; ***R***) in the presence of noise correlations and the information *I*_*ind*_(*S*; ***R***) in the absence of noise correlation. (*I*_*ind*_(*S*; ***R***) is the information obtained replacing *P*_*ind*_(***r***|*s*) for *P*(***r***|*s*) and *P*_*ind*_(***r***|*s*) for *P*(***r***|*s*) in the entropies entering Eq. (1)). *I*_*cor *_quantifies whether the presence of noise correlations increases or decreases the information available in the neural response, compared to the case where such correlations are absent but the marginal probabilities of each element of the response array are the same. *I*_*cor *_can be further broken into two terms *I*_*cor*-*ind *_and *I*_*cor*-*dep*_, as follows:

(6)

*The stimulus independent correlational term, I*_*cor*-*ind*_, reflects the contribution of stimulus-independent correlations. In general, if noise and signal correlations have opposite signs, *I*_*cor*-*ind *_is positive. In this case, stimulus-independent noise correlations increase stimulus discriminability compared to what it would be if noise correlations were absent [17,48]. If, instead, noise and signal correlations have the same sign, *I*_*cor*-*ind *_is negative and stimuli are less discriminable than the zero noise correlation case. In the absence of signal correlation, *I*_*cor*-*ind *_is zero, whatever the strength of noise correlation.

*The stimulus dependent correlational term I*_*cor*-*dep *_is a term describing the impact of stimulus modulation of noise correlation strength. *I*_*cor*-*dep *_is non-negative, and is greater than zero if and only if the strength of noise correlation is modulated by the stimulus. *I*_*cor*-*dep *_was first introduced in Ref. [21] with the name Δ*I*. *I*_*cor*-*dep *_is an upper bound to the information lost by a downstream system interpreting the neural responses without taking into account the presence of correlations [19].

All quantities in the information breakdown can be expressed in terms of the six quantities *H*(***R***), *H*(***R***|*S*), *H*_*lin*_(***R***), *H*_*ind*_(***R***), *H*_*ind*_(***R***|*S*), and *χ*(***R***) where *H*(***R***), *H*(***R***|*S*) were defined above in Eqs. (2,3), and:



The components of the information breakdown can be quantified from the above quantities as follows:



## Implementation

### Computing environment

Our *Information Breakdown Toolbox *(ibTB) has been implemented in Matlab (The Mathworks, Natick, MA) and C taking advantage of Matlab's MEX technology. It has been tested on several platforms (Mac OS X, Windows 32 and 64 bits, Ubuntu Linux) and it can be downloaded, together with a documentation for its installation and its use, from our website [52].

### Data input/output

**Figure 2:**
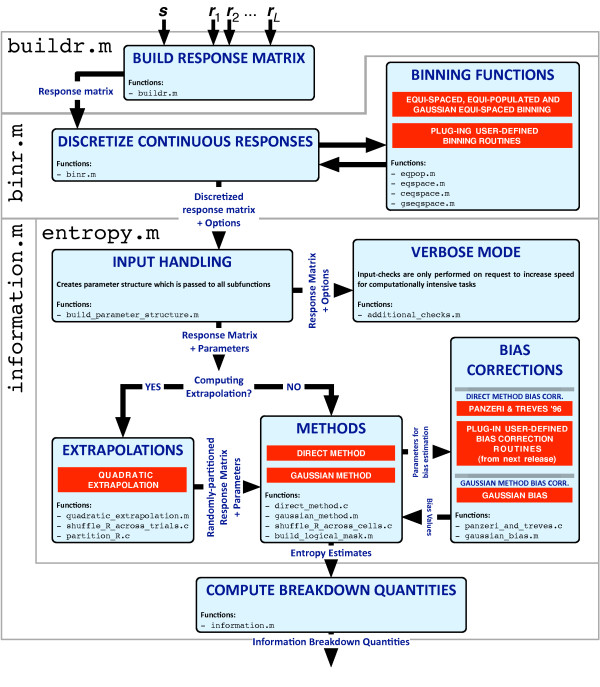
**Structure of the main routines in the ToolBox**. The core function is entropy.m which allows to compute the entropy-like quantities which constitute the building blocks for the calculation of mutual information and of all the other breakdown terms. It is important to note how the modular structure allows for new methods and extrapolation procedures to be easily created and linked to the existing software. In particular, starting with the next release of the toolbox, users will be given the opportunity to easily plug-in their custom bias correction m-files in order to extend the capabilities of the existing code. Other routines in the toolbox are available for the pre-processing of the input to entropy.m. buildr.m allows to build the response-matrix to be fed to the information routines starting from *L *+ 1 -long arrays: the first array stores a list specifying, for each trial, which stimulus was presented to the subject while the *L *remaining arrays specify the *L *corresponding recorded responses. binr.m allows to discretize continuous response-matrices – prior to calls to entropy.m with the the Direct Method – according to a binning method chosen among a list of available binning options. Again, users are given the possibility to define their own binning strategy (in addition to the built-in binning methods) and to easily link their code to the toolbox. Finally, information.m is a wrapper around entropy.m which directly computes the breakdown terms by combining the outputs from this main function, thus skipping all the complex computations necessary for the the calculation of the breakdown quantities.

The main routine in the toolbox is entropy.m (see the workflow of the toolbox in Figure 2) which allows the computation of the fundamental quantities necessary for the computation of the terms which appear in the breakdown decomposition. This routine receives as input a matrix storing the *L *responses to each trial for each stimulus. The user also needs to specify the estimation method, the bias correction procedure and which entropy quantities need to be computed. These can be any of the following: *H*(***R***), *H*(***R***|*S*), *H*_*lin*_(***R***), *H*_*ind*_(***R***), *H*_*ind*_(***R***|*S*), *χ*(***R***) and *H*_*sh*_(***R***), *H*_*sh*_(***R***|*S*) (see below for a definition of these last two quantities).

As shown in Figure 2, two routines are available for the pre-processing of the input to entropy.m. buildr.m allows to build the response-matrix, to be fed to entropy.m, starting from *L *+ 1 -long arrays: the first array stores a list specifying, for each trial, which stimulus was presented to the subject while the *L *remaining arrays specify the *L *corresponding recorded responses. binr.m allows to discretize continuous response-matrices – prior to calls to entropy.m with the the Direct Method – according to a binning method chosen among a list of available binning options. This list includes the *equi-populated *binning (which returns responses whose stimulus-independent probabilities are approximately uniform) and different types of *equi-spaced *discretizations. Users are also given the opportunity to define their own binning strategies by easily linking their binning routine to binr.m (we refer the reader to the documentation to this function for more information on how to define custom binning methods).

Finally, information.m is a wrapper around entropy.m which directly computes the breakdown terms by combining the outputs from this main function. Its input is identical to that of entropy.m except for the list of possible outputs options which can be any or several of the following: *I*(*S*; ***R***), *I*_*sh*_(*S*; ***R***), *syn*, *syn*_*sh*_, *I*_*lin*_, *I*_*sig*-*sim*_, *I*_*cor*_, *I*_*cor*-*sh*_, *I*_*cor*-*ind*_, *I*_*cor*-*dep *_and *I*_*cor*-*dep*-*sh*_.

### Direct Method

The Direct Method [33] for computing information and entropies consists in estimating the probabilities of the discrete neural responses by simply computing the fraction of trials in which each response value is observed, and then by simply plugging into the information and entropy equations the response probabilities estimated in this way. In this subsection, we describe a novel, computationally optimized algorithm for computing the empirical estimates of the probabilities, which is at the core of our toolbox and is the principal reason for its speed.

To describe this algorithm, let's consider, as an example, the calculation of the response probability *P*(***r***). Its direct estimator is given by



where *C*(***r***) is the number of times the response ***r ***has been observed within the total number of available trials . By plugging  into Equation (2), we obtain the direct response-entropy estimator

(7)

The steps required for computing (***R***) according to (7) are the following. First the routine has to run through all of the  available trials and compute the *C*(***r***) values. The program must then loop through all the *N*_*r *_possible ***r ***responses, normalize each *C*(***r***) by  and sum the *C*(***r***) log *C*(***r***) values together:

   for *trial *from 1 to 

      read ***r ***in current trial

      *C*(***r***) → *C*(***r***) + 1

   end loop

   for ***r ***from 1 to *N*_*r*_

      

      

   end loop

A problem with this approach comes from the rapid growing of *N*_*r *_as *L *increases. Let us assume that each element in the response array can take on *M *different values. We have *N*_*r *_= *M*^*L*^. One can thus see that estimating the information quantities according to this two-loops method becomes prohibitive for *N*_*c *_and *M *larger than a few units. However, some simple algorithmic observation can help simplifying the problem significantly. We have

(8)

where ℋ(***R***) = ∑_*r *_*f*(*C*(***r***)) and *f *(·) denotes the function *f*(*x*) = *x *· log *x*.

First of all, Equation (8) suggests that, instead of normalizing each count *C*(***r***) and then summing over ***r***, we can instead first compute ℋ(***R***) and then normalize. This has the advantage of reducing the number of division operations from *N*_*r *_to just one.

Equation (8) tells us even more. Suppose that an additional trial is provided in which the response  is observed. The total number of trials thus increases from  to  + 1. We also need to update the value of *C*() by incrementing it by one. As a result of this change ℋ(***R***) is increased by an amount  given by



This observation suggests that, instead of computing the final value of *C*(***r***) we can update ℋ(***R***) directly at each trial, inside the first loop, therefore skipping the second loop over the *N*_*r *_responses. The procedure for computing (***R***) thus becomes:

   for *trial *from 1 to 

      read ***r ***in current trial

      ℋ(***R***) → ℋ(***R***) + *f*(*C*(***r***) + 1) - *f*(*C*(***r***))

      *C*(***r***) → *C*(***r***) + 1

   end loop

   normalize ℋ(***R***)

where the length of the loop is determined only by the number of available trials.

The previous procedure can be extended to the direct computation of (***R***|*S*), (***R***) and (***R***|*S*). Since the argument of *f*(·) is always an integer, we can store the values computed for *f*(·) and use them for the computation of all four entropic quantities. In the current implementation of the toolbox, the values of *f*(·) persist in memory as long as  does not change. Calls to the toolbox with matrices with the same number of trials perform increasingly faster and the maximum computation speed is achieved when all values of *f*(*x*) for *x *= 1,...,  have been computed.

Finally, let's describe the computation of (***R***) and *χ*^(*d*)^(***R***). Consider a very simple example in which only two responses are recorded (*L *= 2) each of which can only take two possible values 0 and 1. For each stimulus *s *we can thus build the probability arrays



where we used the compact notation *P*_*i*_(*x*|*s*) = *P *(*r*_*i *_= *x*|*s*). If we now wish to build a probability array for *P*_*ind*_(*r*|*s*), as done for the stimulus-conditional response probabilities, we have

(9)

where ***P***_1_(*s*) ⊗ ***P***_2_(*s*) denotes the Kronecker's product between the two probability arrays ***P***_1_(*s*) and ***P***_2_(*s*). Equation (9) can be extended to any *L *and any number of values taken by the responses

(10)

The number of products required to compute ***P***_*ind*_(*s*) and, consequently, also the time required to compute *P*_*ind*_(***r***), (***R***) and *χ*^(*d*)^(**R**), increases rapidly together with the number, *L*, of responses.

Since *L *is not known a priori but it is chosen by the user, in order to minimize the number of multiplications and of iterations while also reducing the overhead due to calls to sub-functions, we implemented Eq. (10) by recursively partitioning the problem into half until pairwise products are reached. For example, for *L *= 6 the routine performs the products in the following order:



### Bias Correction for the Direct Method: plug-in vs bias-corrected procedures

The Direct Method relies on the empirical measure of the response probabilities as histograms of the fraction of trials in which each discrete response value was observed. Naturally, this procedure gives a perfect estimate of the information and entropies only if the empirical estimates of the probabilities equal the true probabilities. However, any real experiment only yields a finite number of trials from which these probabilities have to be estimated. The estimated probabilities are thus subject to statistical error and necessarily fluctuate around their true values. If we just plug the empirical probabilities into the information equations (a procedure often called the "plug-in" procedure in the literature), then the finite sampling fluctuations in the probabilities will lead to a systematic error (bias) in the estimates of entropies and information [31]. In some cases, the bias of the plug-in information estimate can be as big as the information value we wish to estimate. It is therefore crucial to remove this bias effectively in order to avoid serious misinterpretations of neural coding data [31].

Next, we describe and compare four bias correction procedures that we implemented in our toolbox. These procedures, which are among those most widely used in the literature, were selected for inclusion in our toolbox because they are applicable to any type of discretized neural response (whatever its statistics), because they are (in our experience) among the most effective, and because they are guaranteed to converge to the true value of information (or entropy) as the number of trials  increases to infinity.

#### Quadratic Extrapolation (QE)

This bias correction procedure [33] assumes that the bias can be accurately approximated as second order expansions in 1/, that is

(11)

where *a *and *b *are free parameters that depend on the stimulus-response probabilities, and are estimated by re-computing the information from fractions of the trials as follows. The dataset is first broken into two random partitions and the information quantities are computed for each sub-partition individually: the average of the two values obtained (for each quantity) from the two partitions provides an estimate corresponding to half of the trials. Similarly, by breaking the data into four random partitions, it is possible to obtain estimates corresponding to a fourth of the trials. Finally, *a *and *b *are extrapolated as parameters of the parabolic function passing through the /2 and /4 estimates.

#### Panzeri & Treves (PT) Bias Correction

This correction technique computes the linear term *a *in the expansion (11) through an analytical approximation rather than from the scaling of the data of the QE procedure. This approximation depends on the number of response bins with non-zero probability of being observed which is estimated through a bayesian-like procedure. The implementations of this algorithm in our toolbox follows closely the one originally described in Ref. [32].

#### The Shuffling (sh) Procedure

Obtaining unbiased information estimates is particularly challenging when the response array is multidimensional (*i.e. L *> 1), because in this case the number of possible different responses grows exponentially with *L *and it becomes difficult to sample experimentally the response probability. This difficulty arises because, as previously discussed, different elements of the response array are usually correlated. As a consequence, the sampling of the full probability of a response array cannot be reduced to computing the probabilities of each individual array element ("marginal probabilities") as would be legitimate if the response-elements were independent. Fortunately, a technique (called the *shuffling method *[31,36]) can keep the bias introduced by correlations under control, thereby greatly improving our ability to estimate multi-dimensional information. This method [31,36] consists of computing information *I*(*S*; ***R***) not directly through Eq. (1), but through the following formula:

(12)

where *H*_*sh*_(***R***|*S*) is the shuffle noise entropy, *i.e*., the noise entropy computed after randomly permuting, independently for each response, the order of trials collected in response to a stimulus (in other words, it is the noise entropy of the distribution *p*_*sh*_(**r**|*s*)). *I*_*sh*_(*S*; ***R***) has the same value of *I*(*S*; ***R***) for infinite number of trials but has a much smaller bias for finite , owing to the bias cancelation created by the entropy terms in the right hand side of Eq. (12).

It should be noted that, if one is interested in breaking down *I*_*sh*_(*S*; ***R***) (rather than *I*(*S*; ***R***)) into the information components of [17], then *syn*, *I*_*cor *_and *I*_*cor*-*dep *_need to be re-defined as follows:



This three shuffled-corrected quantities, *syn*_*sh*_, *I*_*cor*-*sh *_and *I*_*cor*-*dep*-*sh*_, converge to the same values of their uncorrected counterparts *syn*, *I*_*cor *_and *I*_*cor*-*dep*_, respectively, for infinite number of trials. However the bias of the shuffle-corrected quantities is much smaller when the number of trials is finite. This is especially critical for the computation of *I*_*cor*-*dep *_which is by far the most biased term of the information breakdown [36].

#### Bootstrap Correction

The bootstrap procedure [53,54] consists of pairing stimuli and responses at random in order to destroy all the information that the responses carry about the stimulus. Because of finite data sampling, the information computed using the bootstrapped responses may still be positive. The distribution of bootstrapped information values (over several instances of random stimulus-response pairings) can be used to build a non-parametric test of whether the information computed using the original responses is significantly different from zero. Moreover, the average of the bootstrapped values instances can be used to estimate the residual bias of the information calculation, which can be then subtracted out. The bootstrap evaluation and subtraction of the residual error can be applied to any method to compute information (such as the Direct Method explained above and the Gaussian Method which will be explained below), with or without one of the bias correction procedures described above. In our toolbox, bootstrap estimates can be computed for the quantities *H*(***R***|*S*), *H*_*lin*_(***R***|*S*), *H*_*ind*_(***R***), *H*_*ind*_(***R***|*S*), *χ*(***R***) and *H*_*sh*_(***R***|*S*) from which bootstrapped estimates of *I*(*S*; ***R***) and *I*_*sh*_(*S*; ***R***) are easily obtained. The remaining quantities, *H*(***R***) and *H*_*sh*_(***R***), are not affected by the bootstrapping.

### Gaussian Method

The Direct Method, being based on empirically computing the probability histograms of discrete or discretized neural responses, does not make any assumption on the shape of the probability distributions. This is a characteristic which makes the Direct Method widely applicable to many different types of data.

An alternative approach to the Direct estimation of information is to use analytical models of the probability distributions; fit these distributions to the data; and then compute the information from these probability models. This method has been applied so far relatively rarely in Neuroscience (e.g. [55]). In fact this approach may prove difficult to apply to distributions of spike patterns since in this case appropriate analytical forms of probability distributions are usually not available. However, several situations exist for which it is possible to fit the response distribution to Gaussian functions, especially when dealing with analog brain responses and their transformations, such as Fourier transforms (see the Results section). The Gaussian Method for computing information and entropies is the one based on fitting response probabilities to Gaussian functions.

Under the Gaussian hypothesis, the noise and response entropy and the information are given by simple functions of their variance [2]

(13)

(14)

(15)

where |*σ*^2^(***R***)| and |(***R***)| are the determinants of the matrices of covariance computed across trials and stimuli, and across trials to stimulus *s*, respectively.

Note that the Gaussian Method – which we implemented using a straight computation of variances which are then fed into the above equations – does not necessarily require data discretization prior to the information calculation.

The advantage of the Gaussian Method is that it depends only on a few parameters that characterize the neural response (*i.e*., the variances and covariances of the responses), and is thus more data-robust, and less prone to sampling bias than the Direct calculation. The potential danger with this approach is that the estimates provided by Eq. (15) may be inaccurate if the underlying distributions are not close enough to Gaussians.

Although less severe than in the Direct case, the upward bias of the information calculation due to limited sampling is still present when using the Gaussian Method. When the underlying distributions are Gaussian and when no discretization is used for the responses, an exact expression for the bias of (***R***) and (***R|****S*) can be computed. The result is as follows [56-58]:

(16)

(17)

where *g*_*bias*_(·) is defined as



and *ψ*(·) is the polygamma function (implemented in Matlab as the psi function).

Our toolbox allows the computation of Gaussian estimates without bias correction, with the analytical Gaussian bias correction, Eqs. (16,17), and also with the quadratic extrapolation correction QE. However, using simulated data, we found that quadratic extrapolation did not correct well for bias for the Gaussian Method (results not shown). This can be understood by noting that Eqs. (16,17) indicate that a quadratic data scaling may not necessarily describe well the bias in the Gaussian case.

The Gaussian Method can also be used to compute the terms *I*_*lin*_, *I*_*sig*-*sim *_and *I*_*cor *_of the information breakdown [17] by approximating, *H*_*ind*_(***R***) with *H*_*sh*_(***R***), the response entropy computed after shuffling the neural responses at fixed stimulus. Note that in this case also *H*_*ind*_(***R|****S*) needs to be replaced by *H*_*sh*_(***R|****S*). The calculations of all these quantities is implemented in the Toolbox. Note that the quantity *χ*(***R***) cannot be easily computed with the Gaussian Method, thereby preventing the separation of *I*_*cor *_into *I*_*cor*-*ind *_and *I*_*cor*-*dep*_.

## Results and discussion

In the following we present several case studies and tests of the performance of our ibTB Toolbox on analog neural signals, such as EEGs and LFPs. We emphasize that the toolbox can be effectively applied to spike trains as well as to EEGs and LFPs. The reason why we focus our presentation on EEGs and LFPs is that the very same algorithms implemented in our toolbox have been already illustrated and tested heavily on spike trains; therefore the illustration and test on EEGs and LFPs is more interesting. We however report that we have thoroughly tested our toolbox on spike trains. In particular, we have used our Toolbox to successfully replicate a number of previously published spike train information theoretic studies from our group, reported in Refs. [8,13,14,17,31,36,59].

### Finite sampling bias corrections of information measures from analog neurophysiological signals

We start by testing the performance of bias correction procedures on simulated data. These procedures have been previously tested on simulated spike trains [31], but not yet on analog neural signals. We therefore tested the bias corrections on realistically simulated LFPs whose statistical properties closely matched those of real LFPs recorded from V1 of an anaesthetized macaque in responses to Hollywood color movies presented binocularly to the animal (data from [29]). Details of the simulations procedure are reported in Appendix A. Our goal is to estimate the information (about which of 102 different 2.048 s long movie scenes was presented) carried by the two-dimensional response array made of the power of the simulated LFP at frequencies 4 and 75 Hz. The power in each frequency was discretized into 6 equipopulated bins. Choosing the boundaries of the discretization bins so that each bin is equi-populated is a simple but effective way to obtain high information values even when discretizing an analog signal into a relatively small number of bins. This is because equipopulated binning maximizes the response entropy *H*(***R***) that can be obtained with a given number of response bins [2].

**Figure 3:**
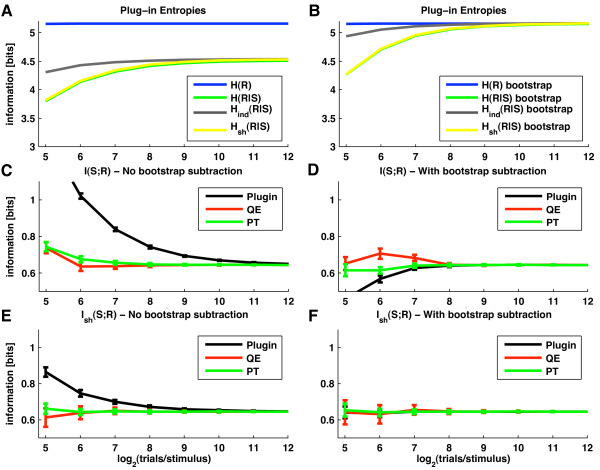
**Comparison of the sampling properties of different information quantities and of bias correction techniques**. The estimates of information and entropies obtained with a number of techniques are tested on simulated data and plotted as a function of the number of generated trials per stimulus. Results were averaged over a number of repetitions of the simulation (mean value ± st. dev. over 50 simulations). We generated simulated LFPs which matched the second order statistics of LFPs recorded from primary visual cortex during visual stimulation with color movies (see Appendix A). The neural response ***r ***used to compute information was a two dimensional response array ***r ***= [*r*_1_, *r*_2_] containing the simulated LFP power at frequencies *f*_1 _= 4 Hz and *f*_2 _= 75 Hz, respectively. The power at each frequency was binned into 6 equi-populated values. **A**: Values of the four plug-in entropies *H*(***R***), *H*(***R***|*S*), *H*_*ind*_(***R***|*S*) and *H*_*sh*_(***R***|*S*). **B**: Values of the bootstrap estimates of the quantities in Panel (A), computed by randomly pairing trials and stimuli. **C**: Mutual information values obtained with plug-in estimate (black line) and following the application of two bias correction procedures, namely QE (red line) and PT (green line). **D**: values of the same quantities reported in panel (C), but after further subtracting the bootstrap correction. **E**: Values of the information *I*_*sh*_(*S*; ***R***) computed using the shuffling procedure with the following bias corrections: plug-in (black line); QE (red line) and PT (green line). **F**: values of the same quantities reported in panel (E), but after further subtracting the bootstrap correction.

In order to illustrate both the origin and magnitude of the bias, it is useful to start the analysis by considering the sampling behavior of the plug-in Direct estimator (we remind that the plug-in estimator is the one that does not uses any bias correction after plugging the empirically estimated probabilities into the information and entropy equations). Figure 3A shows that the plug-in estimates of *H*(***R***|*S*) decrease when decreasing the number of trials. That is, finite sampling makes plug-in entropy estimates biased downward. Intuitively, this can be understood by noticing that entropy is a measure of variability: the smaller the number of trials the less likely we are to fully sample the full range of possible responses and the less variable the neuronal responses appear. Consequently, entropy estimates are lower than their true values [34].

Figure 3A also shows that, in contrast to *H*(***R***|*S*), estimates of *H*(***R***) are essentially unbiased. It is a very common finding that *H*(***R***) is less biased than *H*(***R***|*S*) [31] because the former depends on *P*(***r***) which, being computed from data collected across all 102 stimuli, is better sampled than *P*(***r***|*s*). However, in this specific case, the fact that *H*(***R***) is unbiased stems from the chosen response discretization method: by discretizing each analog LFP power response at the two considered frequencies into 6 equi-populated bins we obtain 36 equi-probable bidimensional responses which provide *H*(***R***) = log_2_(36) ~ 5.17.

Figure 3C shows that the plug-in estimate of information *I*(*S*; ***R***) is biased upward: it tends to be higher than the true value for small number of trials, and then it converges to the true value as the number of trials grows. This upward sampling bias, which originates from the downward sampling bias of *H*(***R***|*S*) (see Eq. (1)), can be as large as the true information value if the number of available trials is low (Figure 3C). Previous research [32] showed that the most crucial parameter in determining the size of the bias for the plug-in estimator is the ratio between the number of trials per stimulus, *N*_*tr*_(*s*), and the cardinality of the response space, which we denote by *N*_*r*_. (In this particular example, the cardinality *N*_*r *_of the two dimensional response space equals 36). The ratio *N*_*tr*_(*s*)/*N*_*r *_tells us how well-sampled are the responses.Figure 3C shows that the plug-in estimator requires *N*_*tr*_(*s*) to be at least two orders of magnitude larger than *N*_*r *_for it to become essentially unbiased, a characteristic that makes this estimator of limited experimental utility. This is why bias correction procedures are needed.

The performance of two such procedures implemented in our Toolbox (PT and QE) is reported in Figure 3C. Both corrections substantially improve the estimates of *I*(*S*; ***R***), which, in this simulation became accurate for *N*_*tr*_(*s*) ≈ 2^7 ^= 128 (i.e. *N*_*tr*_(*s*)/*N*_*r *_≈ 3, to be compared with *N*_*tr*_(*s*)/*N*_*r *_≈ 100 for pure plug-in).

Figure 3D reports the effect of subtracting bias corrections based on data bootstrapping [53,54]. Computing the bootstrap value of the plug-in estimate and then subtracting it from the plug-in information value is not very effective: it leads to a large overestimation of the bias and thus to a big underestimation of the information. The reason why this is the case can be understood by comparing the sampling behavior of the plug-in estimator of *H*(***R***|*S*) before (Figure 3A) and after (Figure 3B) the data bootstrapping. For large numbers of trials, bootstrapping makes *H*(***R***|*S*) equal to *H*(***R***). However, the bias of *H*(***R***|*S*) is much greater after bootstrapping. This is because bootstrapping enhances the support and spread of the stimulus-response distributions *P*(***r***|*s*), which makes these probabilities more difficult to sample [32]. As a consequence the bootstrap estimate of the bias of *I*(*S*; ***R***) is exaggerated and its subtraction from the plug-in estimate of *I*(*S*; ***R***) leads to a severe underestimation of the information. However, if we apply a PT or QE bias correction first, and then estimate and subtract out the remaining residual error by bootstrap, we obtain a far more effective bias removal (Figure 3D). This shows that (consistent with the analytic arguments in [32]), the data bootstrapping gives a good evaluation of the residual bias error after correction, though it overestimates the actual value of the bias per se.

Finally, we considered the effect of computing information through *I*_*sh*_(*S*; ***R***) (Eq. (12) rather than through *I*(*S*; ***R***)). Let us consider first the sampling behavior of the plug-in estimate of the four entropies that make up *I*_*sh*_(*S*; ***R***). Because *H*_*ind*_(***R***|*S*) depends only on the marginal probabilities of the response array, it typically has very small bias (Figure 3A). *H*_*sh*_(***R***|*S*) has the same value of *H*_*ind*_(***R***|*S*) for infinite number of trials, but it has a much higher bias than *H*_*ind*_(***R***|*S*) for finite number of trials. In fact, Figure 3A shows that the bias of *H*_*sh*_(***R***|*S*) is approximately of the same order of magnitude as the bias of *H*(***R***|*S*). Intuitively, this is expected because *P*_*sh*_(***r***|*s*) is sampled with the same number of trials as *P*(***r***|*s*) and from responses with the same dimensionality [21,36]. In this simulation, the biases of *H*_*sh*_(***R***|*S*) and *H*(***R***|*S*) were not only similar in magnitude but actually almost identical (Figure 3A). This, as explained in [36], reflects the fact that for the data simulated here (which represent a low and a high LFP frequency from the same electrode) the correlations among elements of the response array were relatively weak [29] and thus *H*_*sh*_(***R***|*S*) and *H*(***R***|*S*) were very close both in value and sampling properties. Because the biases of *H*_*sh*_(***R***|*S*) and *H*(***R***|*S*) almost cancel each other and since *H*(***R***) is unbiased, the bias of *I*_*sh*_(*S*; ***R***) is almost identical to that of *H*_*ind*_(***R***|*S*). This in turn implies that the plug-in estimator of *I*_*sh*_(*S*; ***R***) must have a much smaller bias than *I*(*S*; ***R***), a fact clearly demonstrated by the results in Figure 3E.

Due to its intrinsically better sampling properties, *I*_*sh*_(*S*; ***R***) has an advantage over *I*(*S*; ***R***) not only when using a plug-in estimation but also when using bias subtraction methods. Figure 3E shows that when using PT or QE corrections, *I*_*sh*_(*S*; ***R***) can be computed accurately even when using only 2^6 ^= 64 trials per stimulus (corresponding to *N*_*tr*_(*s*)/*N*_*r *_≈ 2). When using an additional bootstrapping procedure to subtract the residual bias (Figure 3F), the estimate of *I*_*sh*_(*S*; ***R***) becomes almost completely unbiased, independently of the bias correction used, even with as little as 2^5 ^= 32 trials (corresponding to *N*_*tr*_(*s*)/*N*_*r *_≈ 1).

It should be noted that this behavior applies to cases in which (like the one we simulated) correlations among elements of the response array are relatively weak. In conditions when the correlations among elements of the response array are very strong (as it is often case with both LFPs and spikes recorded from the nearby electrodes), then the sampling behavior of *I*_*sh*_(*S*; ***R***) is still qualitatively similar to that reported here, with the main difference that in cases of stronger correlation *I*_*sh*_(*S*; ***R***) tends to have a small downward (rather than upward) bias. This stems from the fact that in the presence of stronger correlations the bias of *H*_*sh*_(***R***|*S*) tends to be more negative than that of *H*(***R***|*S*) [36], and was verified extensively on simulated spike trains in previous reports [36] and by increasing the level of correlations in these simulated LFPs (results not shown).

In summary, we presented the first detailed test of bias corrections procedures (originally develop for spike trains) on simulated analog neural signals. These simulations (i) confirm that these procedures are effective also on data with statistics close to that of LFPs; (ii) show that in such case it is highly advisable to use *I*_*sh*_(*S*; ***R***) as method to compute information; (iii) indicate that evaluating and subtracting the residual bootstrap errors of *I*_*sh*_(*S*; ***R***) (as done in [29]) is particularly effective. We recommend this procedure to Toolbox users interested in computing information form multidimensional LFP or EEG responses.

### Correlations between different frequency bands of Local Field Potentials in Primary Visual Cortex

In this section we illustrate the Information Breakdown formalism [17] to study whether signal or noise correlations between the LFP powers at different frequencies made them to convey synergistic or redundant information about visual stimuli with naturalistic characteristics. For this study, we analyzed LFPs recorded from the primary visual cortex of anesthetized macaques in response to a binocularly presented naturalistic color movie [29]. Each recording site (51 in total) corresponded to a well-defined V1 visual receptive field within the field of movie projection. From each electrode, we measured LFPs as the 1–250 Hz band-passed neural signal. Each movie was 5 min long and was repeated 40 times in order to sample the probability distribution over the neural responses to each scene. Full details on the experimental procedures are reported in Ref. [29]. The correlation between the LFP activity in different frequency bands on this dataset was studied in Ref. [29] using only linear (Pearson) correlation. Here, we extend these previous results by using the information breakdown, which takes into account both linear and non-linear correlations at all orders [17].

We used the information-theoretic procedure to compute how the power of LFPs at these different frequencies reflects changes in the visual features appearing in the movie. We divided each movie into non-overlapping time windows of length *T *= 2.048 s. Each window was considered as a different "stimulus", *s*, and the corresponding power spectra (obtained in each trial and in each window by means of the multitaper technique [60,61]) were considered as the neural response. From the distribution across trials of the power at each frequency and stimulus window, we computed the mutual information between the stimulus (*i.e*. the considered movie fragment) and the joint power of the LFP at two selected frequencies *f*_1 _and *f*_2_. (Thus, the response ***r ***was a two dimensional array [*r*_1_, *r*_2_] containing the power at frequency *f*_1 _and *f*_2_, respectively.) To compute information, we used the Direct Method together with the shuffled information estimator corrected with the Quadratic Extrapolation bias correction and the bootstrap subtraction (as done in Figure 3F for simulated data).

**Figure 4:**
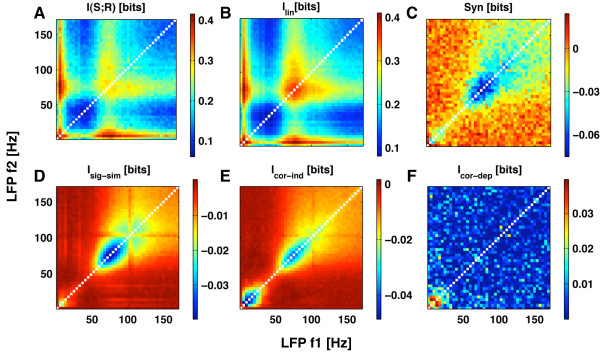
**The information conveyed jointly by V1 LFPs at pairs of frequencies and its breakdown in terms of different correlational components**. The breakdown of the information about naturalistic color movies carried by the power of LFPs at two different frequencies *f*_1 _and *f*_2_. Results are plotted as function of *f*_1 _and *f*_2_, and averaged over a set of 51 recording sites obtained from primary visual cortex of anaesthetized macaques. All estimates were computed using the Direct Method corrected with the QE bias correction procedure and the bootstrap subtraction. Each analog LFP power response was binned into 6 equi-populated values. **A**: The information, *I*(*S*; , ), conveyed jointly about the stimulus by pairs of LFP power responses **B**: The linear sum, *I*_*lin*_(*S*; , ), of the information conveyed independently by each response. **C**: The synergy *syn*(, ), between the responses at the two frequencies. **D**: The signal-similarity term *I*_*sig*-*sim *_**E**: The stimulus-independent noise correlation component *I*_*cor*-*ind *_**F**: the stimulus-dependent correlational component *I*_*cor*-*dep*_.

Figure 4A reports the average over all datasets of the information *I*(*S*; ***R***) carried by the LFP powers about which part of the movie was being presented, as a function of the frequencies *f*_1 _and *f*_2 _at which the power was extracted. Three local maxima of information are present, which involve frequencies either in the low LFP frequency range (below 12 Hz) and in the so-called high-gamma LFP frequency range (60–100 Hz).

The first maximum *I*(*S*; ***R***) occurs when *f*_1 _and *f*_2 _are in the low (below 12 Hz) frequency range. A second, broader maximum is present for *f*_1 _and *f*_2 _in the high gamma range. The highest maximum, however, is obtained when combining a low power response with an high-gamma one. An interesting question is whether the LFP powers belonging to the highly informative low-frequency range and the high-gamma range carry independent or redundant information about the stimuli, and whether any potential redundancy is due to shared sources of variability (*i.e*. noise correlation) or to similarities in the tuning to different scenes of the movie (*i.e*. signal correlation). In the following, we will use the information breakdown to address this question.

Let's first consider any two frequencies *f*_1 _and *f*_2 _belonging to the low frequency range. A comparison of *I*(*S*; ***R***) (Figure 4A) and *I*_*lin *_(Figure 4B) shows that *I*(*S*; ***R***) is only slightly less than *I*_*lin*_. Thus, as made explicit in Figure 4C, there is only a very small negative synergy (*i.e*. a positive redundancy) between low LFP frequencies. To understand the origin of this small redundancy, we used the information breakdown [17]. This formalism shows that low LFP frequencies have little redundancy not because they are independent, but because they share noise correlations whose effect cancel out. In particular, there is a negative stimulus-independent correlation component *I*_*cor*-*ind *_(Figure 4E) which is almost exactly compensated by a stimulus-dependent correlation component *I*_*cor*-*dep *_(Figure 4F). Unlike noise correlations, signal correlations have a very little specific impact on the information carried by the two frequencies (because *I*_*sig*-*sim *_is zero; Figure 4D). These results suggest that the low-frequency LFP bands share a strong common source of variability and thus do not originate from entirely distinct processing pathways, even though they add up independent information about the external correlates.

We then considered the case in which *f*_1 _belongs to the the low frequency range while *f*_2 _is in the high-gamma range. In this case, the powers at *f*_1 _and *f*_2 _added up independent information about the external correlates, because the joint information *I*(*S*; ***R***) (Figure 4A) was equal to *I*_*lin *_(Figure 4B), and as a consequence there is zero synergy between (Figure 4C) between the low and high-gamma LFP frequencies. The information breakdown [17] shows that signal correlations have no impact on the information carried by the two frequencies (because *I*_*sig*-*sim *_is zero; Figure 4D), and that the same applies to both stimulus-dependent and stimulus-independent noise correlations (Figure 4F and 4E). Thus, low LFP frequencies and high-gamma LFPs generated under naturalistic visual stimulation share neither noise nor signal correlations. They appear to be uncorrelated under naturalistic visual stimulation, and are thus likely to arise from fully decoupled neural phenomena.

We finally examined the case in which both *f*_1 _and *f*_2 _belong to the high-gamma frequency range. In this case, there is considerable negative synergy (*i.e*. positive redundancy) between such frequencies. This redundancy can be attributed to signal correlation (because *I*_*sig*-*sim *_is strongly negative), which means that high gamma frequencies have a similar response profile to the movie scenes. The redundancy between high gamma frequencies is further enhanced by a negative effect of stimulus-independent noise correlation *I*_*cor*-*ind*_, Figure 4E. This can be explained by the results of a previously reported linear correlation analysis [29] which suggested a presence of a small amount of positive noise correlation between high gamma frequencies that accompanies the positive signal correlation, and with the fact that a combination of signal and noise correlation with the same sign leads to a negative *I*_*cor*-*ind*_.

It is interesting to note that the results obtained with the information breakdown are compatible with those obtained on the same dataset using a simpler linear signal and noise correlation [29]. Since the information breakdown, unlike the linear correlation theory, is able to capture the impact of non-linear signal and noise correlations if they were present, the equivalence between linear correlation theory and information breakdown can be taken as strong evidence that the linear correlations individuated in [29] are a sufficient description of the functional relationship between LFP responses at different frequencies.

#### Performance of the Gaussian Method

In this section, we illustrate the accuracy and performance on real LFPs responses of the Gaussian Method. We consider again the calculation of how the LFP power encodes information about naturalistic movies, and we use again the same set of LFPs recorded from the primary visual cortex of an anesthetized macaque in response to a binocularly presented naturalistic color movie [29], which were analyzed in the previous section. We computed the information *I*(*S*; ***R***) – about which of the 2.048 s long movie scene in which we divided the movie was being presented – carried by the LFP power at a given frequency *f*. The response ***r ***was a scalar, *r*_*f*_, containing the power at frequency *f*. We estimated the information *I*(*S*; ***R***) either with the Gaussian Method or with the Direct Method.

When using the Gaussian Method, we first estimated the power in each stimulus window and trial using the multitaper technique. We then took the cubic root of this power; we fitted the distribution of this response to each stimulus to a Gaussian; and we finally computed the information through Eqs. (13) and (14) subtracting the analytic gaussian bias correction, Eqs. (16) and (17). The reason for applying the cubic root transformation is that multitaper power estimates are asymptotically chi-square distributed [60] thus their cubic root is approximately Gaussian [62]. The cubic root operation, being monotonic, does not affect the underlying information values of the power, but it makes response probabilities much more Gaussian and thus facilitates information estimation with the Gaussian Method. When using the Direct Method, we simply discretized these transformed power values into *M *equi-populated bins and computed information through Eq. (1) and corrected it for bias using QE. The number of response bins *M *was varied in the range 4–8 (see below).

**Figure 5:**
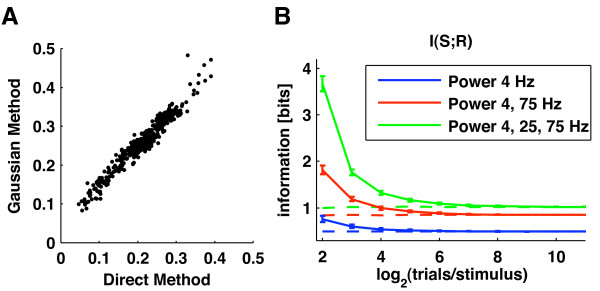
**A comparison of Gaussian and Direct information estimation on V1 LFPs**. **A**: This panel compares the values of information about naturalistic movies carried by V1 LFPs with the Gaussian and the Direct methods. It shows a scatter plot of the information conveyed about the movie by a single LFP frequency computed either with the Gaussian Method, or as a Direct estimate with data discretized into 8 bins. Data were taken from channel 7 of session D04 of the dataset reported in Ref. [29]. Each dot represents an information value obtained at a different LFP frequency with the two techniques considered. This figure shows that, on this dataset, the Gaussian and the Direct calculations provide similar values. The Pearson correlation coefficient between the values computed with the two methods was 0.99. **B**: We tested the Gaussian estimates of information on simulated data. We generated simulated LFPs which matched the second order statistics of LFPs recorded from primary visual cortex during visual stimulation with color movies (see Appendix A). The neural response ***r ***used to compute information was either a one, a two or a three dimensional response array containing the simulated LFP power at frequencies of 4, 25 and 75 Hz. The estimates of the mutual information carried by the power at either one, two or three frequencies were computed using these data and the Gaussian Method and was plotted as function of the generated number of trials per stimulus (mean value ± the standard deviation over 50 simulations). Solid lines and dotted lines represent Gaussian estimates obtained without and with the subtraction of the analytic gaussian bias correction (see text) respectively.

Figure 5A reports a scatter plot of the gaussian information *I*^(*g*) ^and the direct information *I*^(*d*) ^(the latter computed using a discretization with *M *= 8 bins) carried by the power of an example V1 recording channel (electrode 7 in monkey D04) at any given frequency in the range up to 250 Hz. It can be appreciated that the two information estimates are almost identical and are distributed along a line with unitary slope: *I*^(*g*) ^= 1.0 · *I*^(*d*)^. This demonstrates that the Gaussian approximation is extremely precise for the response computed from this dataset, consistent with mentioned properties of multitaper spectral estimators.

A comparison between Gaussian and direct estimates may be useful to evaluate the effect of refining the discretization of neural responses. For this dataset, we found that when using more bins to discretize responses for the Direct Method (*e.g. M *= 10 or *M *= 12 bins), the direct information values *I*^(*d*) ^do not change appreciably (data not shown). However, when decreasing the number of bins to *M *= 4 and *M *= 6 the resulting scatterplot of *I*^(*g*) ^versus *I*^(*d*) ^was again distributed along a line (like in Figure 5A) but with slopes of *I*^(*g*) ^= 1.1 · *I*^(*d*) ^and *I*^(*g*) ^= 1.2 · *I*^(*d*)^, respectively. These findings suggest several conclusions. First, differences that could be observed between gaussian and direct estimates were due to loss of information caused by poor discretization when using low number of bins for the Direct estimate. Second, using 8 response bins is sufficient to capture of the information of the LFP power and using less bins leads to very moderate information losses (of the order of 10–20%). Third, this suggests that knowledge of the second order statistics of the root-transformed power-values is sufficient for extracting the bulk of the information from the LFP power fluctuations.

To demonstrate the sampling properties and data robustness of the Gaussian information estimates, we proceed as we did previously for the Direct Method, generating realistically simulated LFPs whose statistical properties closely matched those of real V1 LFPs (see Appendix A for a description of how data were generated). This time we considered the information about the 102 presented movie sequences carried by the power of either one, two or three different simulated LFP frequencies (*i.e*., the response was, respectively, one, two or three dimensional). Results are reported in Figure 5B. We found that if no bias correction was used, the Gaussian information values were all upward biased, and the bias grew with the dimensionality of the response space (Figure 5B). However, using the analytic bias correction in Eqs. (16) and (17) eliminated the bias completely, with essentially identical accuracy for all considered dimensions.

Taken together, these results indicate that the Gaussian Method can be an extremely accurate and useful tool for studying the information content of analog neural signals. Because of its great data robustness, we strongly recommend its use on any neural signal whose response probabilities are consistent with Gaussian distributions.

### EEGs frequencies encoding visual features in naturalistic movies

We next demonstrate the applicability of our toolbox to the analysis of single-trial EEGs. We considered EEGs recorded from a male volunteer with a 64-channel electrode cap while the subject was fixating the screen during the repeated presentation of a 5 s-long naturalistic color movie presented binocularly. Full details on experimental procedures are reported in Appendix B. We then used our Toolbox to investigate which frequency bands, which signal features (phase or amplitude), and which electrode locations better encoded the visual features present in movies with naturalistic dynamics.

To understand which frequency bands were more effective in encoding the movie, we used a causal bandpass filter (see Appendix B for details) to separate out the range of EEG fluctuations at each electrode into distinct frequency bands (delta: 0.1–4 Hz; theta: 4–8 Hz; alpha: 8–12 Hz; beta: 12–20 Hz). We then extracted, by means of Hilbert transforms of the bandpassed signal, the instantaneous phase and power of the EEG fluctuations in each electrode, frequency band, and trial and examined the time course of amplitude and phase during the movie.

**Figure 6:**
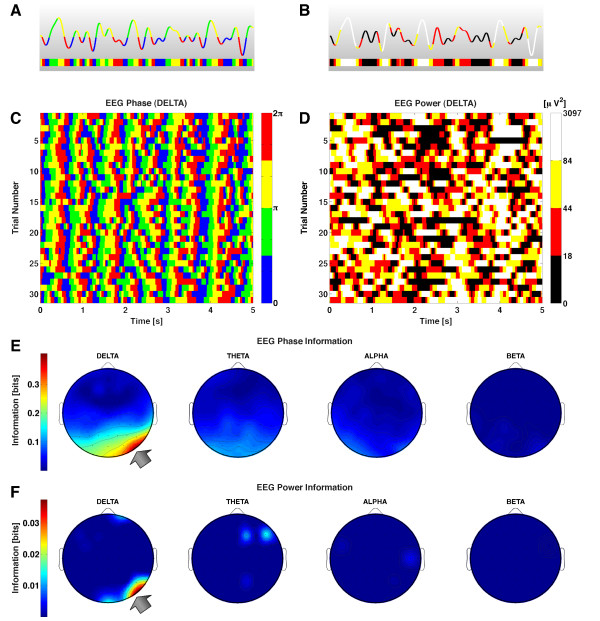
**Information analysis of EEG recordings during vision of naturalistic movies**. The Figure shows EEG responses, and their information, recorded from a human subject watching 5 s-long repeated presentations of a color movie. Data from Panels (A-D) were taken from an example electrode (whose location is reported by the arrow in Panels E-F). **A**: The time course of the phase of the delta-band (0.1 – 4 Hz) EEG during a single trial (*i.e*., a single movie presentation). The single-trial delta-band EEG has been color-coded according to its phase-angle binned into four equally spaced intervals between -*π *and *π*. **B**: The same single-trial delta-band EEG from the example electrode was color-coded according to the instantaneous power binned into four equally probable intervals. **C**: Time course of the instantaneous phases of the 0.1 – 4 Hz (delta) EEG from the example electrode over 30 repetitions of the movie. Phase values were color coded into quadrants exactly as illustrated in Panel (A). **D**: Time course of the binned instantaneous power of the 0.1 – 4 Hz (delta) EEG from the example electrode over 30 repetitions of the movie. Power values were color coded into quadrants exactly as illustrated in Panel (B). **E**: The information conveyed by the binned phase in the delta (0 – 4 Hz), theta (4 – 8 Hz), alpha (8 – 15 Hz) and beta (15 – 30 Hz) frequency bands is plotted topographically across the electrode locations. **F**: The same topographic plot for the EEG instantaneous power information.

Figure 6A shows the EEG response bandpassed in the 0.1–4 Hz (delta) frequency band recorded from electrode PO8 during the 5 s of movie presentation in one randomly selected trial. To visualize how EEG phase was modulated by the movie, we divided the phase range into four equi-spaced quadrants (each spanning a quarter of the oscillation cycle) and labeled each with a different color (Figure 6A). When we considered how the phase changed over repeated trials to the same movie (Figure 6C) it was apparent that the delta phase values were modulated by the movie time, and this modulation was extremely reliable across trials at several times during the movie. This suggests that the delta phases recorded from electrode PO8 carried information about the movie. To quantify this precisely, we computed the mutual information, about which part of the movie was being presented, carried by the EEG delta phase (binned as in Figure 6C). The information calculation was performed by subdividing the time axis into non-overlapping stimulus windows of length *T *= 4 ms, by computing (from the the data plotted in Figure 6C) the probability of phase bins at each different stimulus time, and then using Eq. (1) while correcting for the finite sampling bias with the QE procedure. We found that the information carried by the delta band EEG phase at this electrode was 0.4 bits.

To compare the reliability of phase and power of the delta-range fluctuations at different points of the movie, we discretized the power of the delta band EEG from electrode PO8 at each time point into four equipopulated bins. We found that power was much less reliable across trials than phase (Figure 6D). As a consequence, we also found that power carried only 0.05 bits of information about the movie, and was thus much less informative than the delta phase from the same electrode.

Having illustrated the encoding of the movie by EEGs with an example recording channel and a selected EEG frequency range, we next characterized the behavior across all electrodes and over a wider range of EEG frequencies. Results are plotted in Figure 6E (phase information) and 6F (power information). We found that only low frequency ranges (delta) were informative, and that phase was far more informative than power at all electrodes. This is consistent with the attenuation properties of the skull, which is more likely to attenuate power but with relatively little introduction of phase shifts. The most informative regions were found in the right occipital parietal lobe covering the right visual cortices. It has been hypothesized that such informative low frequency fluctuations observed in visual cortex may reflect the entrainment to slowly changing informative features in the sensory signal [63,64]. To gain some insights on why EEGs recorded from the right hemisphere were far more informative about the movie than those recorded from the left hemisphere, we calculated the mean pixel luminance of the movie clip over the 5 s stimulation window separately for left and right hemifields. We found that the mean luminance was greatest in the left visual field as compared to the right. This provides one potential reason to explain the lateralization of information about the movie observed in this subject.

This example demonstrates the capabilities of the information analysis to extract the most informative components of EEG signals even when using complex dynamic stimulation paradigms and illustrates the potentials of this toolbox for single-trial EEG analysis.

In order to allow users to familiarize with the Toolbox, we have included (as Additional File to this Article) the entire dataset of EEG Delta Phases for all 64 channels and all trials, together with a commented script that loads the data and computes information through the appropriate calls to the Toolbox (Additional file 2: eegtest.zip). Running the script contained in the afore mentioned file outputs the results plotted in top left plot of Fig 6E.

### Comparison with other available toolboxes

Other groups have developed, or are currently developing, toolboxes for the information analysis of neural responses. Here we briefly discuss some of the relative features of current releases of other information theoretic toolboxes, and their complementariness.

Ince and colleagues [65] recently released an information theoretic toolbox for neuroscience data called *pyentropy *based on the Python programming language [66]. This toolbox has the so far unique feature of including an advanced and memory-efficient algorithm for the computation of entropies which are maximal under given constraints, thereby allowing an easy calculation of many of the "maximum entropy" quantities which have received substantial attention in recent years for the study of neuronal interactions [67-69]. The choice of Python as a programming language comes with several advantages provided by its open source nature, its flexibility, and its very efficient use of memory. The use of Python could however be problem for most experimental neuroscience laboratories, which currently make use of Matlab for preprocessing, analysing and plotting the data.

Another available information theoretic toolbox for spike train analysis is the *Spike Train Analysis Toolkit *(*STAToolkit*) [70,71]. *STAToolkit *is, like our toolbox, based on C-MEX technology and like ours can be easily used in Matlab by experimental neuroscience laboratories. A unique and important feature of *STAToolkit *is the large number of estimation methods for the information carried by spike trains, including techniques such as the binless estimation [72] and the metric space approach [73].

With respect to the two above toolboxes, our new toolbox presents two distinctive features. First, it is the only package which has been tested heavily non only on spike trains but also on analog brain recordings such as LFPs, and EEGs. It also includes algorithms which are specific for these signals, such as the Gaussian Method information calculation and its bias correction. The second distinctive features of our toolbox is the speed of computation. This speed advantage is not only due to the C implementation, but also to the new algorithm for fast entropy calculation that we presented here. By comparing systematically the speed of our toolbox on simulated data with the speed of *pyentropy *[65], we found that our toolbox has a speed advantage of typically an order of magnitude to the one of *pyentropy*. We found similar speed advantage in comparison to *STAToolkit*.

### Future Directions

This paper accompanies the first release of ibTB, which we will continue to be developed over the coming years. Some features that we are working to implement in future releases include:

• *Additional bias corrections procedures*. Currently, we implemented some of the best known and most useful bias correction procedures for the computation of mutual information. Other important corrections exist (*e.g*. [34,35]), however, which we plan to implement and include in the toolbox in the near future. Additionally, starting with the next release of ibTB, users will be given the opportunity to plug-in their own custom bias correction routines linking them very easily to the main routines in the toolbox.

• *Additional methods*. The Gaussian Method is one of the many analytical procedures existing for the computation of entropy and mutual information: actually, several other methods are available which take into account other probability distributions [74]. The modular structure of the toolbox allows to very easily add new methods to the toolbox: these will be gradually introduced with future releases.

• fMRI analysis. We are currently in the process of testing and adapting our toolbox to its use with BOLD fMRI data. Although we developed [32] the bias procedure used successfully in recent information analysis of fMRI data some papers [26,75], more work is needed to understand the specific problems caused by the statistics of fMRI data, and how best to use information theory to detect voxels significantly tuned to the stimuli [25]. We plan to report thorough studies of this issues on the toolbox website as soon as possible.

## Conclusion

Neuroscientists can now record, simultaneously and from the same brain region, several types of complementary neural signals, such as spike, LFP, EEG or BOLD responses, each reflecting different and complementary aspects of neural activity at different spatial and temporal scales of organization. A current important challenge of computational neuroscience is to provide techniques to analyze and interpret these data [27,76-81]. We believe that the new fast information theoretic Matlab Toolbox presented here offers a useful technology tool to analyze these complementary brain signals and understand how the brain may combine together the information carried by aspects of neural activity at these different levels of its organization.

## Availability and requirements

• Project name: Information Breakdown ToolBox

• Project home page: 

• Operating system: tested on Mac OS X, Windows 32 and 64 bits, Linux

• Programming language: Matlab (toolbox tested on R2008a and successive releases) and C

• Other requirements: Microsoft Visual C++ 2008 Redistributable Package x86 (or x64) for use on Windows 32 bit (or 64 bit) machine. The package is freely downloadable from Microsoft's website and is only required if Visual C++ is not installed.

• Licence: ibTB is distributed free under the condition that (1) it shall not be incorporated in software that is subsequently sold; (2) the authorship of the software shall be acknowledged and the present article shall be properly cited in any publication that uses results generated by the software; (3) this notice shall remain in place in each source file.

• Any restriction to use by non-academics: none.

## Abbreviations

fMRI: functional magnetic resonance imaging; LFP: Local Field Potential; ibTB: Information Breakdown ToolBox; EEG: Electroencephalogram; BOLD: Blood-oxygenation-level-dependent

## Authors' contributions

CM conceived the fast algorithms, implemented the procedures and wrote the article. KW and VS recorded the EEG data, and commented on the manuscript. NKL recorded the LFP data, and commented on the manuscript. SP supervised the project, co-implemented the procedures and co-wrote the article.

## Appendix A – Simulation of LFP responses

We simulated the LFP power of a recording site in primary visual cortex (V1) in response to many different movie scenes. In brief, data were simulated as follows. We selected from the dataset of [29] a given example recording channel (channel 7 from animal D04), and we computed multitaper estimates of the power at three chosen frequencies (4, 25 and 75 Hz) in response to approximately 2-s-long scenes of Hollywood color movies presented binocularly to the animal. The multitaper technique allows to reduce the variance of the spectral estimates while keeping the bias under control: this is achieved by means of taking the average of different direct spectra computed using tapers which are orthogonal to each other (see [60] for more details). The maximum number of averaged spectra is a free parameter (named *K*) which is set by the user. Here we chose *K *= 3, thereby providing power estimates which are distributed approximately as a chi-square with 6 degrees of freedom. We then applied Wilson and Hilferty's cube-root transformation [62]: this transformation, being monotonic does not affect the information content of the responses while making the response-distributions to a fixed movie scene approximately gaussian (a fact that we also verified empirically). We use the same approach for simulation of multi-dimensional responses, by assuming that the joint distribution of the root-transformed power at two or three different frequencies during each fixed movie scene was a multivariate Gaussian. We generated many instances of this Gaussian power-responses by means of Matlab's mvnrnd function using mean and standard deviation values which were computed, for each scene, from the real data. For entropy estimates computed using the Direct Method, the data simulated in this way have been further discretized into 6 equi-populated response bins.

## Appendix B – Methods of EEG recording during presentations of short naturalistic movies

The EEG was acquired using a 64 channel electrode cap (BrainAmp MR, BrainProducts). Electrode placement followed the International 10–20 System and electrodes were all referenced to a frontal central electrode (FCz). Electrode impedances were kept below 15 KOhms. Horizontal and vertical eye movements were recorded using an electro-oculogram (EOG) with electrodes placed over the outer canthus of the left eye as well as below the right eye. Subjects were comfortably seated in a dimly lit room. EEG recordings were digitally recorded at 1000 Hz with a bandpass of 0.1–250 Hz and stored for offline analysis. A small fixation cross on black background was shown in order to indicate the beginning of the trial. After 2 seconds of fixation, a 5 second movie segment (full field) was presented, followed by 2 seconds of continued fixation, resulting in trials totaling 9 seconds of fixation. A movie clip, consisting of fast moving and colorful scenes from a commercially available movie, was presented 50 times. All data analysis procedures were implemented with the Matlab programming language in combination with the EEGlab analysis toolbox [82] as described below. Postprocessing was performed using the EEGlab analysis software (Neuroscan). EEG epochs (-1000 to 5000 ms temporal range) were created based on the onset of triggers recorded during the recording session. An EOG artifact correction algorithm was used to remove all trials with amplitudes that exceed ± 75 mV. After artifact rejection, 30 movie presentation trials remained.

To obtain bandpassed EEGs from each electrode, we bandpassed the raw EEG signal sampled at 1 KHz with a zero-phase-shift Kaiser filter with sharp transition bandwidth (1 Hz), very small passband ripple (0.01 dB), high stopband attenuation (60 dB), and bandwidth corresponding to the considered band (*e.g*. 0.1–4 Hz for delta; 4 – 8 Hz for theta, etc. – see main text). These filters were exactly equal to those used for LFPs in Refs [13,14]; we refer to these references for more details.

## Supplementary Material

Additional file 1 **Information Breakdown ToolBox**. This file contains the m-files and executables which constitute the Information Breakdown Tool-Box (ibTB). The most up-to-date version of the code can be also obtained at the following site: . Click here for file

Additional file 2 **EEG_TEST**. This file includes the entire dataset of EEG Delta Phases for all 64 channels and all trials, together with a commented script that loads the data and computes information through the appropriate calls to the Toolbox: running the script outputs the results plotted in top left plot of Fig 6E. Click here for file
